# Supplementary material for: Structure and mechanism of biosynthesis of Streptococcus mutans cell wall polysaccharide
Source: Nat Commun. 2025 Jan 22;16:954. doi: 10.1038/s41467-025-56205-1 (PMC11754754; doi:10.1038/s41467-025-56205-1)
Supplement: Supplementary file 1 — Supplementary Information [file 41467_2025_56205_MOESM1_ESM.pdf]

Supplementary information for:

Structure and mechanism of biosynthesis of *Streptococcus mutans* cell wall polysaccharide

Jeffrey S. Rush[1], Svetlana Zamakhaeva[2], Nicholas R. Murner[2], Pan Deng[3, 7], Andrew J. Morris[4, 8], Cameron W. Kenner[2], Ian Black[5], Christian Heiss[5], Parastoo Azadi[5], Konstantin V. Korotkov[1], Göran Widmalm[6], and Natalia Korotkova[1,2*]

[1]Department of Molecular and Cellular Biochemistry, University of Kentucky, Lexington, Kentucky, USA

[2]Department of Microbiology, Immunology and Molecular Genetics, University of Kentucky, Lexington, Kentucky, USA

[3]Department of Pharmaceutical Sciences, College of Pharmacy, University of Kentucky, Lexington, Kentucky, USA

[4]Division of Cardiovascular Medicine and the Gill Heart Institute, University of Kentucky, Lexington, Kentucky, USA

[5]Complex Carbohydrate Research Center, University of Georgia, Athens, Georgia, USA

[6]Department of Organic Chemistry, Arrhenius Laboratory, Stockholm University, Stockholm, Sweden

[7]Present address: Jiangsu Key Laboratory of Neuropsychiatric Diseases and College of Pharmaceutical Sciences, Soochow University, Suzhou, Jiangsu, China

[8]Present address: Department of Pharmacology and Toxicology, University of Arkansas for Medical Science and Central Arkansas Veterans Affairs Healthcare System, Little Rock, Arkansas, USA

*Correspondence and request for materials should be addressed to N.K. (email: nkorotkova@uky.edu)

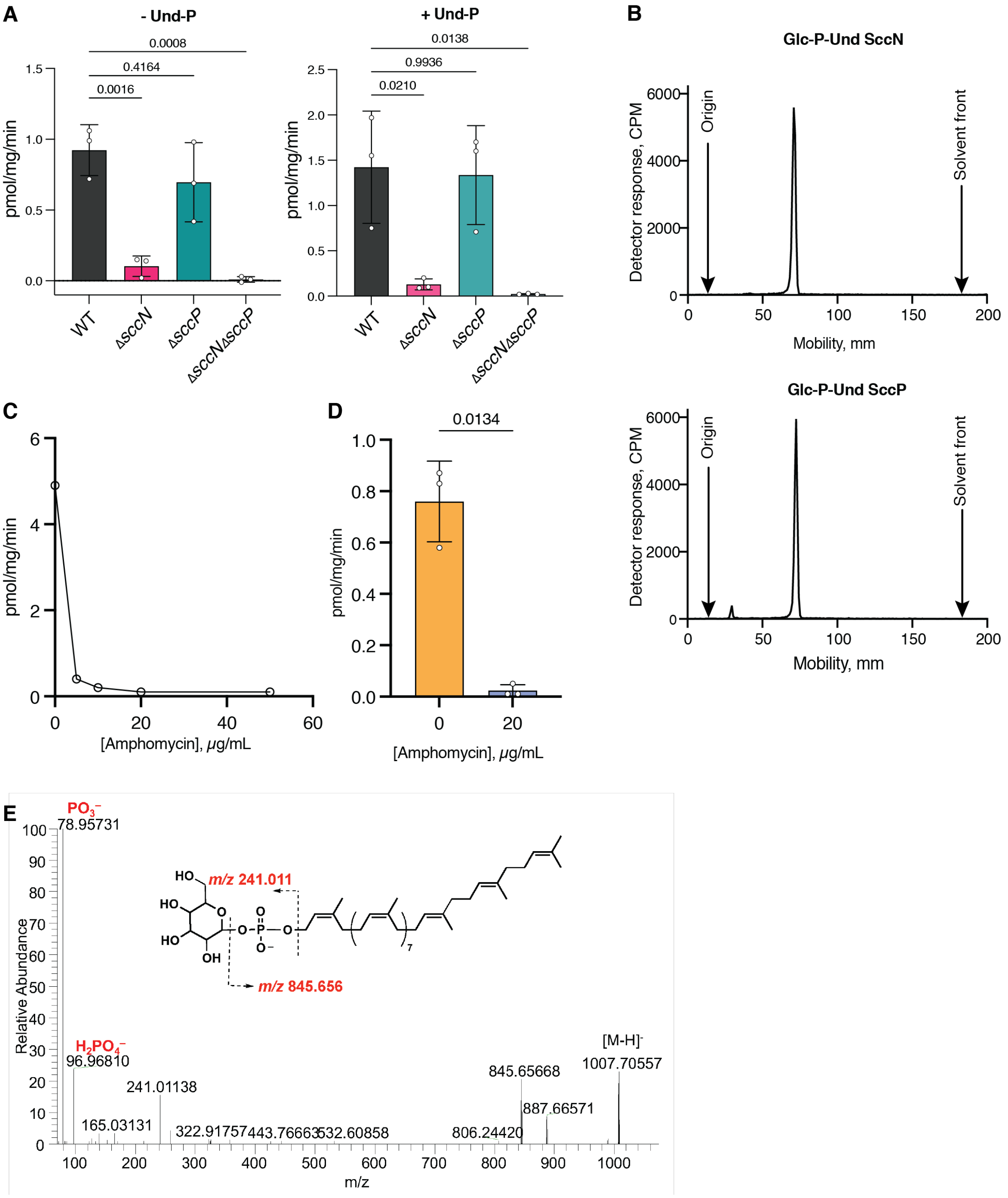

Supplementary Fig. 1. SccN and SccP synthesize Glc-P-Und.

(**a**) Membrane fractions from various *S. mutans* strains were assayed for Glc-P-Und synthase activity in the presence and absence of 20 μM Und-P, added as a sonicated dispersion in 1 % CHAPS, as described in Methods. The results are averages from

three independent membrane preparations ± S.D. *P* values were calculated by one-way ANOVA with Tukey's multiple comparison test. (**b**) Thin layer chromatography of [^{3}H] Glc-P-Unds prepared *in vitro* from enzymatic reactions containing solubilized membrane proteins of *E. coli* JW2347 cells expressing either SccN or SccP. Approximately 10,000 cpm of [^{3}H] Glc-P-Und, from reactions catalyzed by either SccN or SccP, as described in Methods, were spotted on 5 x 20 cm glass-backed Analtech Silica Gel HL sheets, resolved by ascending thin layer chromatography developed in $CHCl_3/CH_3OH/H_2O/NH_4OH$ (65:30:4:1) and detected using a Bioscan AR2000 radiochromatoscanner. Figure is a representative image of two independent experiments. (**c**) Concentration-dependent inhibition of Glc-P-Und synthesis by the addition of amphomycin. Membrane fractions from *S. mutans* Xc were incubated with UDP-[^{3}H]Glc and the indicated concentrations of amphomycin and assayed for the formation of [^{3}H]Glc-P-Und as described in Methods, except that 1 mM $CaCl_2$ was included to facilitate the function of the antibiotic. The experiment was performed independently two times and yielded the same results. Representative image from one experiment is shown. (**d**) Membrane fractions from three independent isolates of *S. mutans* Xc were tested, *in vitro*, for Glc-P-Und synthase activity in the presence and absence of 20 μg/mL amphomycin. Two-tailed unpaired t-test with Welch's correction was used to determine *P* values. (**e**) ESI-MS analysis of compound(s) co-migrating with synthetic [^{3}H]Glc-P-Und purified from the *S. mutans* membrane fraction by preparative TLC. Figure is a representative image of two independent experiments.
Source data for **a**, **c** and **d** are provided as a Source data file.

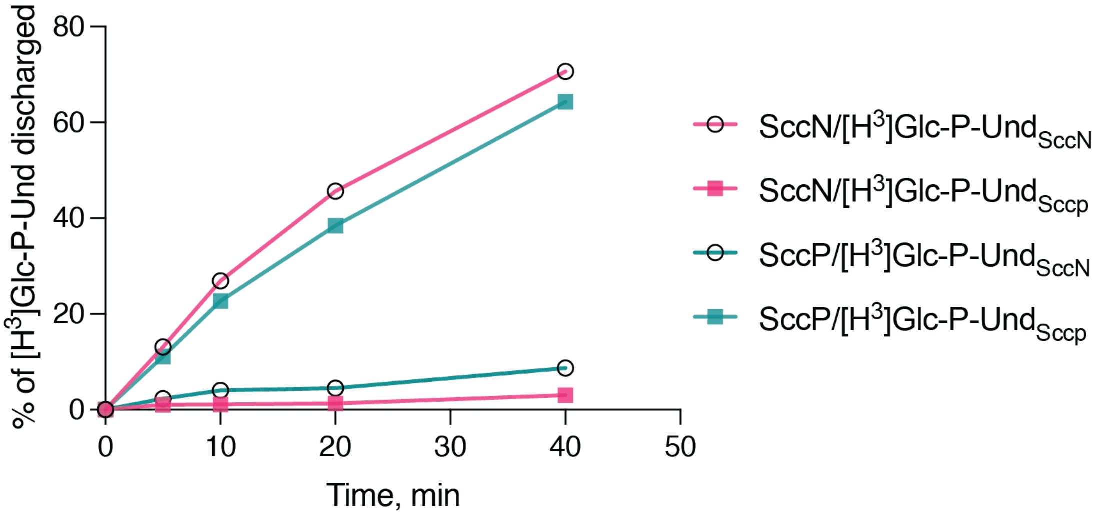

Supplementary Fig. 2. Time-dependent reverse reactions of SccN and SccP to reform UDP-Glc from their respective enzymatic products and UDP.

[^{3}H]Glc-P-Und$_{SccN}$, synthesized by SccN, and [^{3}H]Glc-P-Und$_{SccP}$, synthesized by SccP, were tested as substrates in the discharge reactions, containing soluble, partially-purified SccN or SccP, as described in Methods. The experiment was performed independently three times and yielded the same results. Representative image from one experiment is shown. Source data are provided as a Source data file.

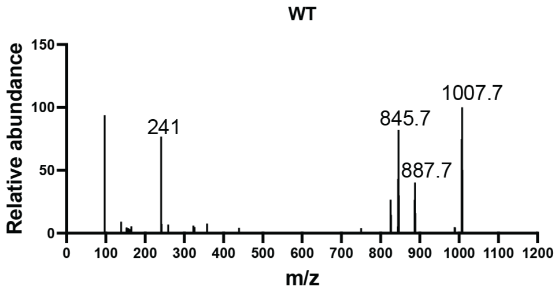
WT
Relative abundance
m/z
241
845.7
887.7
1007.7
0
50
100
150
0 100 200 300 400 500 600 700 800 900 1000 1100 1200

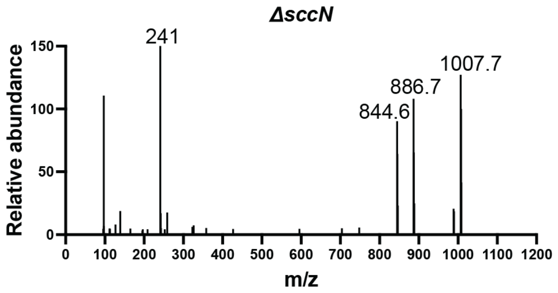
ΔsccN
Relative abundance
m/z
241
844.6
886.7
1007.7
0
50
100
150
0 100 200 300 400 500 600 700 800 900 1000 1100 1200

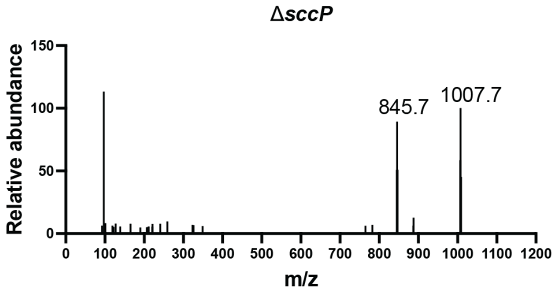
ΔsccP
Relative abundance
m/z
845.7
1007.7
0
50
100
150
0 100 200 300 400 500 600 700 800 900 1000 1100 1200

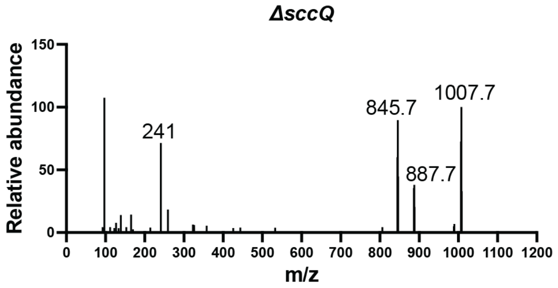
ΔsccQ
Relative abundance
m/z
241
845.7
887.7
1007.7
0
50
100
150
0 100 200 300 400 500 600 700 800 900 1000 1100 1200

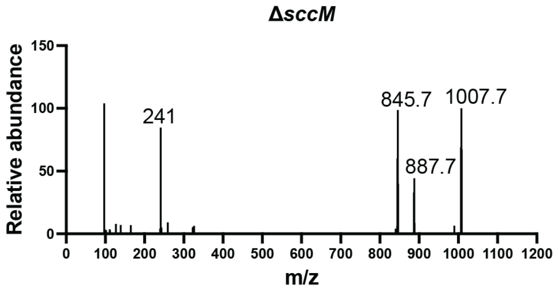
ΔsccM
Relative abundance
m/z
241
845.7
887.7
1007.7
0
50
100
150
0 100 200 300 400 500 600 700 800 900 1000 1100 1200

Supplementary Fig. 3. ESI-MS/MS analysis of Glc-P-Unds purified from *S. mutans* WT, Δ*sccN*, Δ*sccP*, Δ*sccM* and Δ*sccQ* strains by preparative TLC.

Phospholipids were extracted with chloroform/methanol, deacylated in KOH/methanol, purified by preparative TLC and analyzed by Q-Exactive Orbitrap LC/MS as described in Methods. The experiment was performed independently two times and yielded the same results. A representative image from one experiment is shown. Source data are provided as a Source data file.

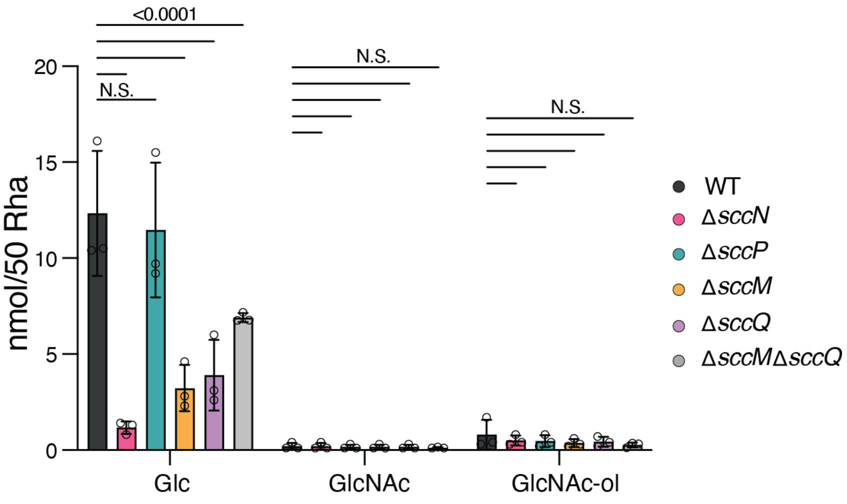

Supplementary Fig. 4. Glycosyl content of SCCs derived from various deletion mutants of *S. mutans.*

SCCs were released from cell wall preparations from the indicated mutant strains of *S. mutans* by mild acid hydrolysis, chemically reduced with sodium borohydride, partially purified by SEC on Biogel P150 and analyzed for glycosyl composition by gas chromatography/mass spectrometry as trimethylsilyl derivatives of *O*-methyl glycosides, as described in Methods. Moles of each sugar from three independent preparations were normalized to a hypothetical polymer containing 50 Rha residues. *P* values were calculated by two-way ANOVA with Dunnett’s multiple comparison test. Source data are provided as a Source Data file.

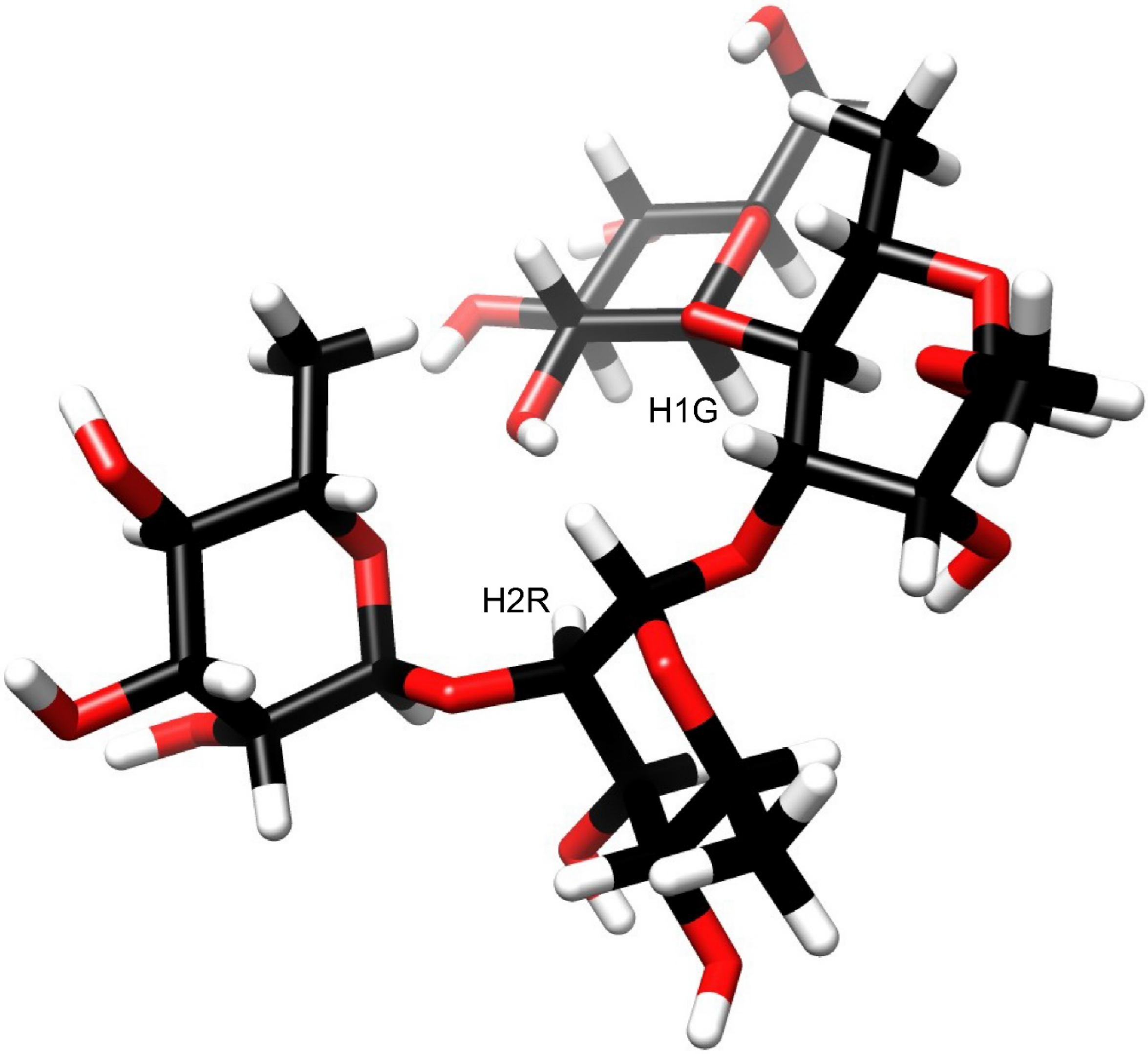

Supplementary Fig. 5. Molecular model made by CarbBuilder [10] and graphically presented using UCSF Chimera [11] of the tetrasaccharide α-L-Rha*p*-(1→2)-α-L-Rha*p*-(1→3)[β-D-Glc*p*-(1→4)]-α-L-Rha*p*-OMe representing a branched (3,4-Rha) region in the SCC.

The anomeric proton (H1G) of the β-D-Glc*p*-(1→4)-linked side-chain residue and the H2 proton (H2R) of the 2-linked rhamnosyl residue in the backbone show a mutual NOE in ^{1}H,^{1}H-NOESY NMR spectrum of the SCC due to spatial proximity, which is substantiated by the model.

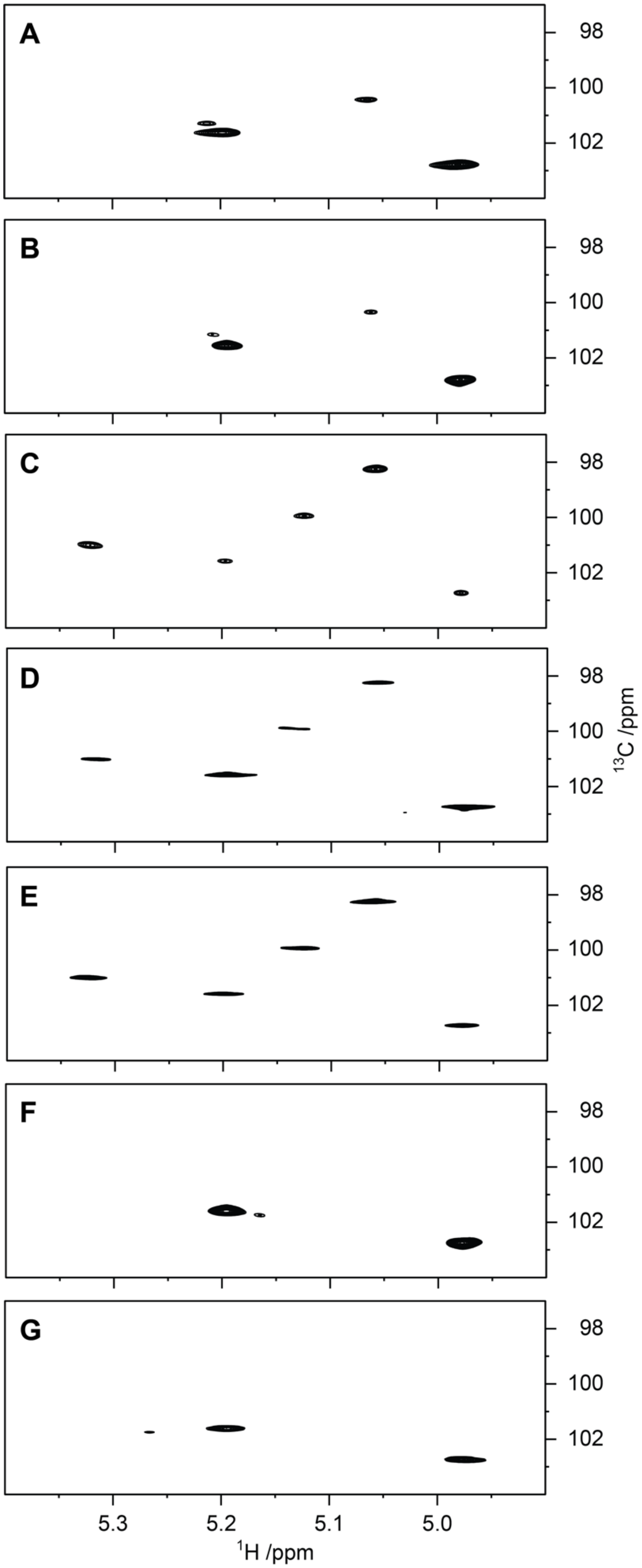
A
B
C
D
E
F
G
98
100
102
13C /ppm
5.3
5.2
5.1
5.0
1H /ppm

Supplementary Fig. 6. Selected region for anomeric resonances in ^{1}H,^{13}C-HSQC NMR spectra of the SCC variants isolated from various deletion mutants of *S. mutans.*

SCC variants were isolated from purified cell wall of *S. mutans* strains, as described in Methods, and subjected to ^{1}H,^{13}C-HSQC NMR. The region containing anomeric resonances from individual preparations is shown. (**a**) Δ*sccM*Δ*sccQ*, (**b**) Δ*sccM*, (**c**) Δ*sccP*, (**d)** Δ*sccQ*, (**e**) WT, (**f**) Δ*sccN*, and (**g**) Δ*sccN*Δ*sccP*.

CASPER report

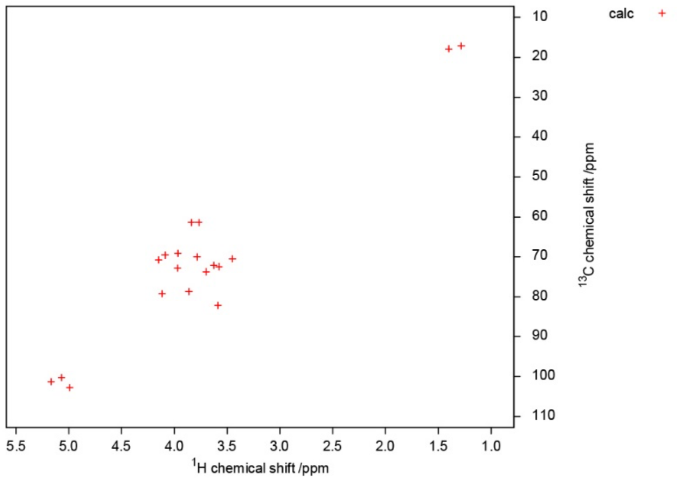

Predicted ^{13}C and ^{1}H NMR chemical shifts

Structure

→2) [α-D-Glc^{iii} (1→4)]α-L-Rha^{ii} (1→3) α-L-Rha^{i} (1→

→3) α-L-Rha^{i} (1→	1	2	3	4	5	6	
Expected Calc. Error: 3.06	102.76	70.81	78.75	72.19	70.05	17.20	
	4.99	4.15	3.86	3.63	3.79	1.28	
→2,4) α-L-Rha^{ii} (1→	1	2	3	4	5	6	
Expected Calc. Error: 2.69	101.31	79.29	69.63	82.22	69.15	17.91	
	5.17	4.12	4.09	3.59	3.97	1.40	
α-D-Glc^{iii} (1→	1	2	3	4	5	6	6
Expected Calc. Error: 0.17	100.29	72.48	73.76	70.52	72.86	61.41	
	5.07	3.58	3.70	3.45	3.97	3.77	3.84

Generated 2024-01-12 14:48:36+01:00.

Supplementary Fig. 7. NMR chemical shift prediction by CASPER [12] of the repeating unit structure containing the α-D-Glc*p*-(1→4)-linked side-chain residue, detected in the Δ*sccM* SCC.

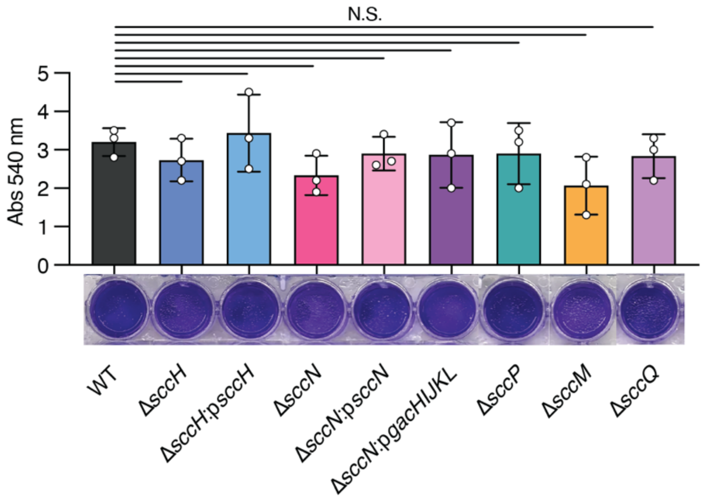

Supplementary Fig. 8. Exopolysaccharide-based biofilms produced by *S. mutans* strains.

To obtain biofilms, *S. mutans* strains were incubated in UFTYE medium supplemented with 1% (wt/vol) sucrose for 24 h at 37 °C in presence of 5% CO_2. Biofilms were analyzed as outlined in Methods. Image of crystal violet stained-biofilms (bottom panel) is a representative image of three independent experiments. Quantification of biofilm formation is shown in top panel. Columns and error bars represent the mean and S.D., respectively (n = 3). Data were analyzed by one-way ANOVA with Dunnett's multiple comparisons test. No significant differences were observed. Source data are provided as a Source Data file.

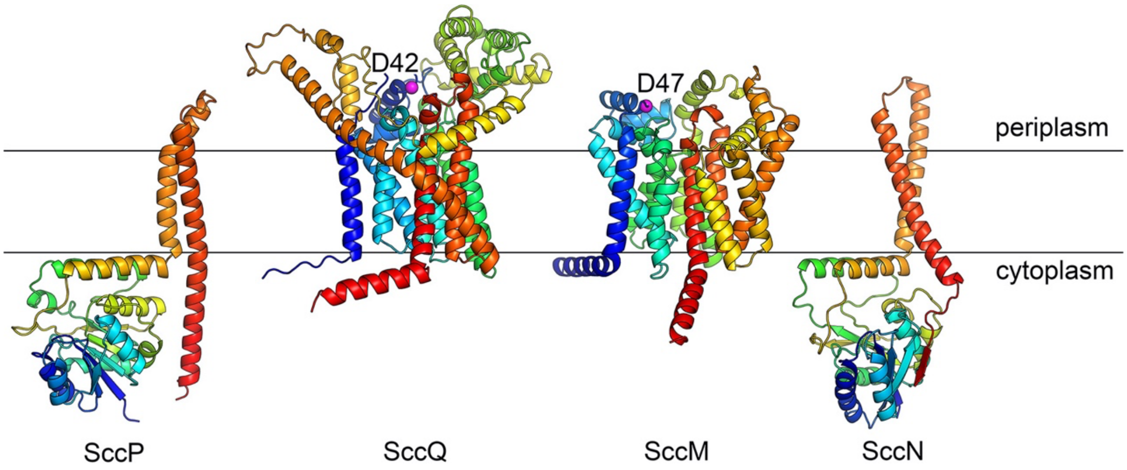

Supplementary Fig. 9. Proposed topological orientation of glycosyltransferases involved in the glucosylation of *S. mutans* SCC.

Structural models predicted by AlphaFold2 pipeline [13, 14] for *S. mutans* SccP, SccQ, SccN and SccM are shown as cartoon diagrams colored in rainbow colors from N-terminus (blue) to C-terminus (red), relative to the bacterial plasma membrane. Magenta spheres highlight the key active-site residues of SccQ and SccM. The membrane topology was predicted using TopCons [15].

Supplementary Table 1. Activity of membrane fractions of *E. coli* strains, carrying empty expression vector, vector with *sccN* or vector with *sccP* in presence of Und-P and UDP-[^{3}H]Glc [a]

Gene expressed	Activity, pmol/mg
None	0.4
sccN	603.6
sccP	278.0

[a] Reaction mixtures contained 50 mM Tris-Cl, pH 7.4, 20 mM $MgCl_2$, 2 mM ATP, 2 mM sodium orthovanadate, 10 mM 2-mercaptoethanol, 15 μM Und-P, dispersed ultrasonically in 1 % CHAPS, 0.35 % CHAPS (final concentration in the reaction), 5 μM UDP-Glc (1600 cpm/pmol) and 20-60 μg solubilized *E coli* membrane proteins (expressing *sccN* or *sccP* on a plasmid or carrying empty expression vector) in a total volume of 0.02 mL. Following 3 min at 37 °C reactions were stopped by the addition of 2 mL $CHCl_3/CH_3OH$ and the [^{3}H]glucolipid product isolated as described in Methods. The data are average rates from incubations and are representative of more than three independent experiments.

Supplementary Table 2. Glycosyl linkages detected in rhamnopolysaccharides from various strains of *S. mutans* determined as partially methylated alditol acetates [a]

Glycosyl linkage[b]	WT			Mean±SD	Δ*sccN*			Mean±SD	Δ*sccP*			Mean±SD	Δ*sccQ*			Mean±SD	Δ*sccM*			Mean±SD	Δ*sccM*Δ*sccQ*			Mean±SD
	mol/mol				mol/mol				mol/mol				mol/mol				mol/mol				mol/mol			
t-Rha	0.1	0.3	0.2	0.2±0.1	0.3	0.8	0.4	0.5±0.3	0.1	0.4	0.2	0.2±0.2	0.3	0.7	0.4	0.4±0.2	0.2	0.5	0.3	0.3±0.2	0.1	0.1	0.0	0.1±0.0
2-Rha	20.9	19.2	22.9	21±1.9	22.8	21.7	23.5	22.7±0.9	19.7	17.3	22.5	19.9±2.6	22.0	20.2	23.3	21.8±1.6	16.9	16.8	17.9	17.2±0.6	18.8	13.8	17.4	16.7±2.5
3-Rha	10.4	10.2	9.2	9.9±0.6	23.5	21.9	22.2	22.5±0.8	9.8	10.5	8.9	9.7±0.8	19.2	18.0	18.3	18.5±0.6	24.3	21.5	22.9	22.9±1.4	19.2	18.2	14.8	17.4±2.3
3,4-Rha	0.2	0.3	0.1	0.2±0.1	3.2	4.8	3.4	3.8±0.9	0.0	0.1	0.0	0.1±0.1	0.1	0.2	0.0	0.1±0.1	1.8	2.5	1.8	2.1±0.4	0.1	0.4	0.1	0.2±0.2
2,3-Rha	14.7	14.7	14.3	14.6±0.3	0.2	0.5	0.3	0.3±0.2	16.8	16.6	15.4	16.3±0.8	7.8	9.4	7.1	8.1±1.2	0.2	0.4	0.3	0.3±0.1	0.2	0.6	0.1	0.3±0.3
2,4-Rha	1.8	2.5	1.6	2±0.5	0.1	0.3	0.2	0.2±0.1	3.4	4.8	2.9	3.7±1.0	0.7	1.5	0.8	1±0.4	6.7	8.2	6.8	7.2±0.9	7.7	11.0	13.4	10.7±2.8
2,3,4-Rha	1.9	2.7	1.8	2.1±0.5	n.d.*	n.d.*	n.d.*		n.d.*	0.1	n.d.*		n.d.*	0.1	n.d.*		n.d.*	0.1	n.d.*		0.0	0.2	0.1	0.1±0.1
t-Glc	20.5	17.2	19.2	19±1.7	3.4	5.2	3.3	4±1.1	20.0	17.5	19.1	18.8±1.3	8.3	9.2	6.9	8.1±1.1	8.6	9.7	7.7	8.7±1.0	3.8	4.4	3.2	3.8±0.6
t-GlcNAc	0.1	0.6	0.3	0.3±0.2	0.1	0.5	0.4	0.3±0.2	0.1	1.0	0.3	0.5±0.5	0.1	0.7	0.3	0.4±0.3	0.1	0.4	0.3	0.3±0.2	0.1	0.5	0.4	0.3±0.2
4-GlcNAcOL	0.3	0.8	0.4	0.5±0.3	0.2	0.8	0.5	0.5±0.3	0.2	1.2	0.3	0.6±0.6	0.3	0.9	0.3	0.5±0.4	0.1	0.6	0.4	0.4±0.2	0.0	0.8	0.5	0.5±0.4

[a] Partially methylated alditol acetates were prepared from purified rhamnopolysacchrides, detected by GC-MS and normalized to a polymer containing an average of 50 Rha units, as described in Methods. *n.d. indicates none detected. Moles of each linkage type per molecule were calculated by multiplying the mol % for each component sugar by the relative area percent for each linkage type found in the gas chromatogram and then normalized to a hypothetical polymer containing 50 Rha units. Individual values of each linkage type from three separate analyses, as well as average and standard deviation calculations, are shown. Source data are provided as a Source data file.

[b] Proposed glycosyl structures of linkage abbreviations:

2-Rha	→2)-α-L-Rha*p*-(1→
3-Rha	→3)-α-L-Rha*p*-(1→
2,3-Rha	→2)-α-L-Rha*p*-(1→3)[α-D-Glc*p*-(1→2)]-α-L-Rha*p*-(1→
2,4-Rha	→3)-α-L-Rha*p*-(1→2)[α-D-Glc*p*-(1→4)]-α-L-Rha*p*-(1→
3,4-Rha	→2)-α-L-Rha*p*-(1→3)[β-D-Glc*p*-(1→4)]-α-L-Rha*p*-(1→
2,3,4-Rha	→2)-α-L-Rha*p*-(1→3)[α-D-Glc*p*-(1→2)][β-D-Glc*p*-(1→4)]-α-L-Rha*p*-(1→

Supplementary Table 3. Cell size analysis [a]

S. mutans strain	Cell #	Cell length, μm±SD	Cell width, μm±SD
WT	144	0.86±0.11	0.63±0.10
Δ*sccQ*	198	0.76±0.10	0.66±0.07

[a] DIC images were used to determine cell sizes of *S. mutans* strains. Cell size (μm) was measured by ImageJ, ObjectJ plugin, and the results were analyzed by GraphPad Prism 9.3. using unpaired two-tailed *t*-test with Welch correction. Total number of cells was n = 144 for WT and n = 198 for Δ*sccQ*. Values are reported with standard deviation. Source data are provided as a Source data file.

Supplementary Table 4. Bacterial strains and plasmids

Strain or plasmid	Description [a]	Reference
Streptococcus mutans		
Xc	Serotype *c* strain, wild-type (WT)	[1]
Δ*sccH*	*sccH* deletion mutant (has a nonpolar erythromycin resistance cassette inserted in *sccH*), Erm^R	[2]
Δ*sccH*:p*sccH*	Δ*sccH* is complemented with p*sccH* carrying WT *sccH,* Erm^R, Cam^R	[2]
Δ*sccN*	*sccN* deletion mutant (has a nonpolar spectinomycin resistance cassette inserted in *sccN*), $Spec^R$	[3]
Δ*sccN*:p*sccN*	Δ*sccN* is complemented with p*sccN* carrying WT *sccN,* $Spec^R$, Cam^R	[3]
Δ*sccN*:p*gacHIJKL*	Δ*sccN* is complemented with p*gacHIJKL*. $Spec^R$, Cam^R	[3]
Δ*sccP*	*sccP* deletion mutant (has a nonpolar spectinomycin resistance cassette inserted in *sccP*), $Spec^R$	[3]
Δ*sccM*	*sccM* deletion mutant (has a nonpolar spectinomycin resistance cassette inserted in *sccM*), $Spec^R$	This study
Δ*sccQ*	*sccQ* deletion mutant (has a nonpolar spectinomycin resistance cassette inserted in *sccQ*), $Spec^R$	This study
Δ*sccN*Δ*sccP*	*sccN sccP* double-gene deletion mutant (has nonpolar spectinomycin and erythromycin resistance cassettes inserted in *sccN* and *sccP,* respectively), $Spec^R$ Erm^R	[3]
Δ*sccM*Δ*sccQ*	*sccM sccQ* double-gene deletion mutant (has nonpolar erythromycin and spectinomycin resistance cassettes inserted in *sccM* and *sccQ,* respectively), $Spec^R$ Erm^R	This study
Δ*sccN*Δ*sccQ*	*sccN sccQ* double-gene deletion mutant (has nonpolar kanamycin and spectinomycin resistance cassettes inserted in *sccN* and *sccP,* respectively), Kan^R $Spec^R$	This study
Δ*sccM*Δ*sccN*	*sccM sccN* double-gene deletion mutant (has nonpolar erythromycin and kanamycin resistance cassettes inserted in *sccM* and *sccN,* respectively), Erm^R Kan^R	This study
Δ*sccM*Δ*sccP*	*sccM sccP* double-gene deletion mutant (has nonpolar erythromycin and spectinomycin resistance cassettes inserted in *sccM* and *sccP,* respectively), Erm^R $Spec^R$	This study
Escherichia coli		

DH5α	*E. coli* cells used for cloning	Invitrogen
JW2347	*E. coli* K-12 strain BW25113 [4] with a deletion of the *gtrB* gene [5]	[6]
Plasmids		
pBAD33_SccN	A pBAD33 derived plasmid expressing *sccN,* Amp^R, Cam^R	This study
pBAD33_SccP	A pBAD33 derived plasmid expressing *sccP,* Amp^R, Cam^R	This study
pLR16T	Vector encoding spectinomycin resistance cassette. $Spec^R$	[7]
pOSKAR	Vector encoding kanamycin resistance cassette. Kan^R	[8]
pHY304	Vector encoding erythromycin resistance cassette. Erm^R	[9]

[a] Antibiotic resistance markers: Erm^R, erythromycin; Kan^R, kanamycin; $Spec^R$, spectinomycin; Cam^R, chloramphenicol, Amp^R, ampicillin

Supplementary Table 5. Primers used for construction of bacterial mutants and plasmids

Primer	Sequence [a,b]	Genetic manipulations
Smu.833-f	GGTTCTGACAGTCGTCTCTC	*sccN* deletion with a nonpolar kanamycin resistance cassette
Kan-Smu.833-r1	**CAGTATTTAAAGATACC**GGTTTCTTCCTCATTATAAC	
Smu.833-Kan-f1	TAATGAGGAAGAAACC**GGTATCTTTAAATACTGTAG**	
Kan-Smu.833-f2	**TGAATTGTTTTAGTAC**GATTTACAGGATCCGCCAG	
Smu.833-Kan-r2	CGGATCCTGTAAATC**GTACTAAAACAATTCATCCAG**	
Smu.833-r	GCAACAAAATTTAGAATCAACAAC	
SccM-f	GTGGGACTTAATGTTAGTG	*sccM* deletion with a nonpolar spectinomycin resistance cassette
Spec-SccM-r1	**CACTATTTTGGTCGAC**CAATAGGCGGTAATGATTC	
SccM-Spec-f1	GAATCATTACCGCCTATTG**GTCGACCAAAATAGTGAGGAGG**	
Spec-SccM-f2	**AAAATTATAAGGATCC**GAGATCAAACGATCTTTGCG	
SccM-Spec-r2	CGCAAAGATCGTTTGATCTC**GGATCCTTATAATTTTTTAATCTG**	
SccM-r	CTGATACCTAACTTATTTATAATG	
SccM-f	GTGGGACTTAATGTTAGTG	*sccM* deletion with a nonpolar erythromycin resistance cassette
Erm-SccM-r1	**CATCTAATTTAACTTCAATTCC**CAATAGGCGGTAATGATTC	
SccM-Erm-f1	GAATCATTACCGCCTATTG**GGAATTGAAGTTAAATTAGATG**	
Erm-SccM-f2	**CGGGAGGAAATAATTCTATG**GAGATCAAACGATCTTTGCG	
SccM-Erm -r2	CGCAAAGATCGTTTGATCTC**CATAGAATTATTTCCTCCCG**	
SccM-r	CTGATACCTAACTTATTTATAATG	
SccQ-f	GTTAATCACCTTTACCAAGG	*sccQ* deletion with a nonpolar spectinomycin resistance cassette
Spec-SccQ-r1	**CACTATTTTGGTCGAC**GGCTATTCGAAAAATTCCAATG	
SccQ-Spec-f1	TTTTTCGAATAGCC**GTCGACCAAAATAGTGAGGAGG**	
Spec-SccQ-f2	**AAAATTATAAGGATCC**GCTATTTCAATTGCTTCAGG	
SccQ-Spec-r2	GCAATTGAAATAGC**GGATCCTTATAATTTTTTAATCTG**	
SccQ-r	GCTAATTCGAAAGCTTTTCG	
SccQ-f	GTTAATCACCTTTACCAAGG	*sccQ* deletion with a nonpolar erythromycin
Erm-SccQ-r1	**CATCTAATTTAACTTCAATTCC**GGCTATTCGAAAAATTCCAATG	

SccQ-Erm-f1	TTTTTCGAATAGCC**GGAATTGAAGTTAAATTAGATG**	resistance cassette
Erm-SccQ-f2	**CGGGAGGAAATAATTCTATG**GCTATTTCAATTGCTTCAGG	
SccQ-Erm -r2	GCAATTGAAATAGC**CATAGAATTATTTCCTCCCG**	
SccQ-r	GCTAATTCGAAAGCTTTTCG	
sccM-check-f	GATTCTTTGACATTCGATAAATC	Verification of Δ*sccM*
sccM-check-r	GATGTCCTGACATAAACATCG	
sccQcheck-f	GAGGGAGATTGGTCTAATTG	Verification of Δ*sccQ*
sccQcheck-r	CAGCTGAGTTAGAGCAAGAAAAAG	
sccN-XbaI-f	GCGACTCTAGACCAATTATTAATTTTCAAGG	Construction of pBAD33_SccN
sccN-HindIII-r	CGCGCAAGCTTCCTATAGCCTTTATCCTTTTTC	
SccP-XbaI-f	GCGACTCTAGAAGGAGAATTTATACTATGACAGAG	Construction of pBAD33_SccP
SccP-SalI-r	CGCTGCGTCGACCCTAAAAACTATTTACGGCC	

[a] Restriction sites are underlined.

[b] Extensions complementary to the antibiotic resistance cassettes are in bold.

Supplementary Table 6. Spontaneous mutations detected in the mutants of *S. mutans* Xc by whole-genome sequencing

Strain	Target insertion of antibiotic resistance cassette[a]	Spontaneous mutations[a]	Annotation
Δ*sccH*	Smu.831	n.d.[b]	
Δ*sccM*	Smu.832	n.d.	
Δ*sccN*	Smu.833	n.d.	
Δ*sccP*	Smu.834	Smu.82<br>V113I (GTT→ATT)	DnaK
		Smu.1237c<br>D12E (TAT→TGT)	Nuclear transport factor 2 family protein
Δ*sccQ*	Smu.835	n.d.	
Δ*sccM*Δ*sccN*	Smu.832<br>Smu.833	n.d.	
Δ*sccM*Δ*sccQ*	Smu.832<br>Smu.835	Smu.834<br>Y37C (TAT→TGT)	SccP
Δ*sccN*Δ*sccP*	Smu.833<br>Smu.834	n.d.	
Δ*sccN*Δ*sccQ*	Smu.833<br>Smu.835	n.d.	
Δ*sccM*Δ*sccP*	Smu.832<br>Smu.834	Smu.82<br>V113I (GTT→ATT)	DnaK
		Smu.1237c<br>D12E (TAT→TGT)	Nuclear transport factor 2 family protein

[a] corresponds to genes in *S. mutans* UA159 (GenBank: AE014133.2)

[b] n.d. indicates none detected.

Supplementary References

1. Koga T, Asakawa H, Okahashi N, Takahashi I. Effect of subculturing on expression of a cell-surface protein antigen by *Streptococcus mutans*. *Journal of general microbiology* **135**, 3199-3207 (1989).

2. Edgar RJ*, et al.* Discovery of glycerol phosphate modification on streptococcal rhamnose polysaccharides. *Nature chemical biology* **15**, 463-471 (2019).

3. Zamakhaeva S*, et al.* Modification of cell wall polysaccharide guides cell division in Streptococcus mutans. *Nature chemical biology* **17**, 878-887 (2021).

4. Datsenko KA, Wanner BL. One-step inactivation of chromosomal genes in Escherichia coli K-12 using PCR products. *Proc Natl Acad Sci U S A* **97**, 6640-6645 (2000).

5. Liu B*, et al.* Structure and genetics of Escherichia coli O antigens. *FEMS Microbiol Rev* **44**, 655-683 (2020).

6. Baba T*, et al.* Construction of Escherichia coli K-12 in-frame, single-gene knockout mutants: the Keio collection. *Mol Syst Biol* **2**, 2006 0008 (2006).

7. Rajagopal L, Vo A, Silvestroni A, Rubens CE. Regulation of purine biosynthesis by a eukaryotic-type kinase in Streptococcus agalactiae. *Mol Microbiol* **56**, 1329-1346 (2005).

8. Le Breton Y, McIver KS. Genetic manipulation of *Streptococcus pyogenes* (the Group A Streptococcus, GAS). *Current protocols in microbiology* **30**, Unit 9D 3 (2013).

9. Chaffin DO, Beres SB, Yim HH, Rubens CE. The serotype of type Ia and III group B streptococci is determined by the polymerase gene within the polycistronic capsule operon. *J Bacteriol* **182**, 4466-4477 (2000).

10. Kuttel MM, Stahle J, Widmalm G. CarbBuilder: Software for building molecular models of complex oligo- and polysaccharide structures. *J Comput Chem* **37**, 2098-2105 (2016).

11. Pettersen EF*, et al.* UCSF Chimera--a visualization system for exploratory research and analysis. *J Comput Chem* **25**, 1605-1612 (2004).

12. Dorst KM, Widmalm G. NMR chemical shift prediction and structural elucidation of linker-containing oligo- and polysaccharides using the computer program CASPER. *Carbohydrate research* **533**, 108937 (2023).

13. Jumper J*, et al.* Highly accurate protein structure prediction with AlphaFold. *Nature* **596**, 583-589 (2021).

14. Varadi M*, et al.* AlphaFold Protein Structure Database in 2024: providing structure coverage for over 214 million protein sequences. *Nucleic Acids Res* **52**, D368-D375 (2024).

15. Tsirigos KD, Peters C, Shu N, Kall L, Elofsson A. The TOPCONS web server for consensus prediction of membrane protein topology and signal peptides. *Nucleic Acids Res* **43**, W401-407 (2015).
