# Supplementary material for: Structure and mechanism of biosynthesis of Streptococcus mutans cell wall polysaccharide
Source: Nat Commun. 2025 Jan 22;16:954. doi: 10.1038/s41467-025-56205-1 (PMC11754754; doi:10.1038/s41467-025-56205-1)
Supplement: Supplementary file 2 — Reporting Summary [file 41467_2025_56205_MOESM2_ESM.pdf]

nature portfolio

Corresponding author(s): Natalia Korotkova

Last updated by author(s): 12/09/2024

Reporting Summary

Nature Portfolio wishes to improve the reproducibility of the work that we publish. This form provides structure for consistency and transparency in reporting. For further information on Nature Portfolio policies, see our Editorial Policies and the Editorial Policy Checklist.

Statistics

For all statistical analyses, confirm that the following items are present in the figure legend, table legend, main text, or Methods section.

n/a	Confirmed	
☐	☒	The exact sample size (*n*) for each experimental group/condition, given as a discrete number and unit of measurement
☐	☒	A statement on whether measurements were taken from distinct samples or whether the same sample was measured repeatedly
☐	☒	The statistical test(s) used AND whether they are one- or two-sided<br>*Only common tests should be described solely by name; describe more complex techniques in the Methods section.*
☒	☐	A description of all covariates tested
☐	☒	A description of any assumptions or corrections, such as tests of normality and adjustment for multiple comparisons
☐	☒	A full description of the statistical parameters including central tendency (e.g. means) or other basic estimates (e.g. regression coefficient) AND variation (e.g. standard deviation) or associated estimates of uncertainty (e.g. confidence intervals)
☐	☒	For null hypothesis testing, the test statistic (e.g. *F*, *t*, *r*) with confidence intervals, effect sizes, degrees of freedom and *P* value noted<br>*Give P values as exact values whenever suitable.*
☒	☐	For Bayesian analysis, information on the choice of priors and Markov chain Monte Carlo settings
☒	☐	For hierarchical and complex designs, identification of the appropriate level for tests and full reporting of outcomes
☒	☐	Estimates of effect sizes (e.g. Cohen's *d*, Pearson's *r*), indicating how they were calculated

Our web collection on statistics for biologists contains articles on many of the points above.

Software and code

Policy information about availability of computer code

Data collection	The NMR spectra were recorded on a Bruker AVANCE III 700 MHz spectrometer equipped with a 5 mm TCI Z-Gradient Cryoprobe (1H/13C/15N) and an AVANCE NEO 400 MHz NMR spectrometer equipped with a 5 mm probe.<br>GC-MS analysis of the TMS methyl glycosides and partially methylated alditol acetates (PMAAs) was performed on an Agilent 7890A GC interfaced to a 5975C MSD employing electron impact ionization. The inlet was set to 250 °C and helium was used as a carrier gas. For the composition analysis, separation was achieved using a 30 meter Equity-1 CG capillary column. For linkage analysis, separation was achieved using a 30 meter SP-2330 CG capillary column.<br>Mass-spectrometry data of phospholipids were collected using Thermo Xcalibur 4.0 (Thermo Fisher Scientific, Inc.).<br>Scanning electron microscopy images were obtained using an FEI Helios Nanolab 660 dual beam system. Differential interference contrast (DIC) microscopy images were obtained using Leica SP8 equipped with 100X, 1.44 N.A. objective.
Data analysis	Statistical analysis was performed using GraphPad Prism version 9.2.0 and 9.5.1.<br>The NMR spectra were processed, analyzed and plotted using TopSpin 4.1.4 software (Bruker BioSpin).<br>Glycosyl composition and linkage data were analyzed using Agilent ChemStation software version B.04.03.<br>Thermo Tracefinder and Compound Discoverer software versions 5.1 and 3.3 were used to analyze mass-spectrometry data of phospholipids.<br>The Qual Browser and Quan Browser functions in Xcalibur 4.0 have been used for processing of mass-spectrometry data of phospholipids.<br>The sizes of cells were analyzed using ImageJ software 1.54f with ObjectJ plugin 1.05i.

For manuscripts utilizing custom algorithms or software that are central to the research but not yet described in published literature, software must be made available to editors and reviewers. We strongly encourage code deposition in a community repository (e.g. GitHub). See the Nature Portfolio guidelines for submitting code & software for further information.

Data

Policy information about availability of data

All manuscripts must include a data availability statement. This statement should provide the following information, where applicable:

- Accession codes, unique identifiers, or web links for publicly available datasets
- A description of any restrictions on data availability
- For clinical datasets or third party data, please ensure that the statement adheres to our policy

All data generated during this study are included in the article and Supplementary information files. The authors have no restriction on data availability

Research involving human participants, their data, or biological material

Policy information about studies with human participants or human data. See also policy information about sex, gender (identity/presentation), and sexual orientation and race, ethnicity and racism.

Reporting on sex and gender	n/a
Reporting on race, ethnicity, or other socially relevant groupings	n/a
Population characteristics	n/a
Recruitment	n/a
Ethics oversight	n/a

Note that full information on the approval of the study protocol must also be provided in the manuscript.

Field-specific reporting

Please select the one below that is the best fit for your research. If you are not sure, read the appropriate sections before making your selection.

- [x] Life sciences
- [ ] Behavioural & social sciences
- [ ] Ecological, evolutionary & environmental sciences

For a reference copy of the document with all sections, see nature.com/documents/nr-reporting-summary-flat.pdf

Life sciences study design

All studies must disclose on these points even when the disclosure is negative.

Sample size	No statistical methods were used to determine a sample size, because the sample size was not a factor of the analysis for the experiments. All sample sizes were determined in accordance with published literature relevant to a particular experiment (see for example van Hensbergen, V. P. et al. 2018, PLoS Pathog 14(10):e1007348 and Edgar, R. J. et al. 2019 Nature Chemical Biology 15, 463-471) and they were optimal to generate statistically significant results. In general, the experimental analysis was performed at least in triplicate unless otherwise indicated.
Data exclusions	No data was excluded from analyses
Replication	All replicates were performed in independent measurements, in different days and similar results were obtained. All experiments were conducted at least three times.
Randomization	No randomization was applied to other experiments because the study does not impose a treatment on a group of objects or subjects. Furthermore, selection bias is not relevant to this study because no animals or human subjects were involved, and uncontrollable conditions did not affect the results of the experiments.
Blinding	Blinding was used for microscopy analysis. Investigator was blinded to the group allocation of bacterial cell.

Reporting for specific materials, systems and methods

We require information from authors about some types of materials, experimental systems and methods used in many studies. Here, indicate whether each material, system or method listed is relevant to your study. If you are not sure if a list item applies to your research, read the appropriate section before selecting a response.

Materials & experimental systems

n/a	Involved in the study
☒	☐ Antibodies
☒	☐ Eukaryotic cell lines
☒	☐ Palaeontology and archaeology
☒	☐ Animals and other organisms
☒	☐ Clinical data
☒	☐ Dual use research of concern
☒	☐ Plants

Methods

n/a	Involved in the study
☒	☐ ChIP-seq
☒	☐ Flow cytometry
☒	☐ MRI-based neuroimaging

Plants

Seed stocks	n/a
Novel plant genotypes	n/a
Authentication	n/a
